# Tetralogy of Fallot with an Anomalous Course of the Brachiocephalic Vein

**DOI:** 10.21470/1678-9741-2023-0047

**Published:** 2023-07-18

**Authors:** Larissa Alves Leite Matos, Isaac Azevedo Silva, Fernando Cesar Gimenes Barbosa Santos, Ulisses Alexandre Croti

**Affiliations:** 1 CardioPedBrasil, Centro do Coração da Criança, Hospital da Criança e Maternidade de São José do Rio Preto - Fundação Faculdade Regional de Medicina de São José do Rio Preto/Faculdade de Medicina de São José do Rio Preto, São José do Rio Preto, São Paulo, Brazil

**Keywords:** Tetralogy of Fallot, Brachiocephalic Veins, Ventricular Heart Septal Defects, Echocardiography, Bundle Branch Block

## Abstract

Clinical data: Infant, 11-month-old, male, diagnosis of Tetralogy of Fallot with retrotracheoesophageal course of the brachiocephalic vein. Usual findings of Tetralogy of Fallot on physical examination. Technical description: Chest radiography showed slightly reduced pulmonary vascular markings and no cardiomegaly. Normal preoperative electrocardiogram with postoperative right bundle branch block. Usual findings of Tetralogy of Fallot on echocardiogram. Postoperative computed tomography angiography confirmed left brachiocephalic vein with anomalous retrotracheoesophageal course, configuring a U-shaped garland vein, in addition to postoperative findings of total correction of Tetralogy of Fallot. Operation: Complete surgical repair was performed with pulmonary valve commissurotomy and placement of bovine pericardial patch to solve right ventricular outflow tract obstruction, pulmonary trunk enlargement, and ventricular septal defect closure. Comments: Systemic venous drainage may show variations in patients with Tetralogy of Fallot. These abnormalities are usually of little clinical relevance, as they are asymptomatic. We presented a rare case of retrotracheoesophageal course of an anomalous left brachiocephalic vein with intraoperative diagnosis, confirmed by imaging during postoperative follow-up, without compromising clinical management or surgical approach.

## CASE PRESENTATION

### Clinical Data

Male, 11-month-old, weight: 7.7 Kg, height: 0.72 m, referred to our service diagnosed with Tetralogy of Fallot (TOF), presenting classic episodes of cyanotic spells. From a rural area in the Northern region of Brazil; due to lack of congenital heart service in this location, he waited several months for surgical intervention.

Physical examination showed an ejection systolic murmur at left sternal border. Regular pulse at 112 bpm and pulse oximetry with 92% oxygen saturation. Remaining physical findings were unremarkable.

## TECHNICAL DESCRIPTION

### Chest Radiography

Visceral and thoracic *situs solitus*. Cardiothoracic ratio of 0,53. Slightly reduced pulmonary vascular markings.

### Electrocardiography

Sinus rhythm with normal axis for age (S QRS + 100°), PR interval of 120 ms, QRS of 80 ms, and QTc of 424 ms. After surgical repair, patient developed right bundle branch block.

### Echocardiography

*Situs solitus* in levocardia, usual venoatrial, atrioventricular, and ventriculoarterial connections. Malalignment of conal septum with a large perimembranous ventricular septal defect, extending to the outlet portion of the right ventricle, typical of TOF. Atrial septal defect of 12 mm.

Hyperthrophic right ventricle with significant subpulmonary and valvar pulmonary stenosis, outlet right ventricle, and pulmonary valve peak and medium gradient were 95 mmHg and 60 mmHg, respectively.

Pulmonary valve with thickened semilunar valves and commissural fusion measuring 7.0 mm (Z score: -0.59), with normal-sized pulmonary trunk and pulmonary arteries branches. Aortic valve dilatation of 14.2 mm (Z score: +4.95). Normal biventricular function.

### Computed Tomography Angiography

**Fig. 1:**
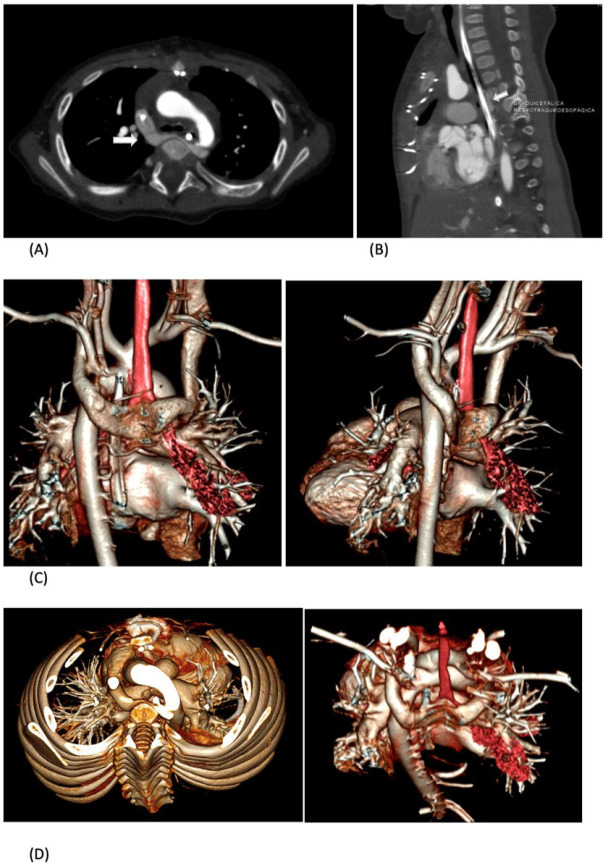
Contrast-enhanced computed tomography showing axial (A) and sagittal (B) images of the U-shaped retroesophageal course of left brachiocephalic vein (white arrow) which joins the right brachiocephalic vein to form the superior vena cava. Volume rendered image as seen from posterior aspect (C) and view from the top (D) shows abnormal course of left brachiocephalic vein into U- shaped vein. (red: trachea).

*Situs solitus* in levocardia. Systemic venous return through the vena cava to the right atrium with slightly increased dimensions. Confirmation of observed finding during surgical repair of left brachiocephalic vein (LBCV) with anomalous retrotracheoesophageal course, configuring a U-shaped garland vein (Figures 1 A to D). Narrowing of left main bronchus at crossing level with the anomalous brachiocephalic vein.

Postoperative findings of complete TOF repair with interventricular communication closure with patch, signs of enlargement of right ventricular outflow tract with pulmonary valve/subvalvular region narrowing, dilatation of the left pulmonary artery, and aorta at the sinus of Valsalva level.

Supra-aortic branches anatomical variant (1^st^ branch: common origin of right and left common carotid arteries; 2^nd^ branch: left vertebral artery origin directly from aortic arch; 3^rd^ branch: left subclavian artery).

### Operation

Patient was scheduled for complete TOF repair. In the operating room, after standard median sternotomy, no LBCV was found, but a vertical left vein resembling left vena cava (LVC). However, there was no connection between this LVC and the heart. Therefore, surgical dissection was extended through retroaortic region to retrotracheoesophageal space. Finally, this LBCV came to join the superior vena cava, configuring the U-shaped garland vein.

Next, procedure was performed as planned, with bicaval and aortic cannulation. Right ventricular outflow tract was relieved by myotomy. Pulmonary valve was corrected by commissurotomy. Ventricular septal defect closed with bovine pericardial patch. Right ventricular outflow tract and pulmonary trunk were enlarged with bovine pericardial patch. And atrial septal defect was closed with a bovine pericardial patch.

A transesophageal echocardiogram after cardiopulmonary bypass removal showed good results.

## COMMENT

The spectrum of systemic venous anomalies varies widely and is usually asymptomatic. This case is an anomalous course of an LBCV, with intraoperative diagnosis.

The normal course of the left brachiocephalic (innominate) vein is from left to right, anterior to the aortic arch in the superior mediastinum, joining the right brachiocephalic (innominate) vein forming the right superior vena cava.

The anomalous LBCV (ALBCV) was first described by Kerschener in 1888^[1]^. Webb et al.^[2]^ described computed tomographic findings in 1982. In a study published in 2010, the incidence of ALBCV was 0.02% in > 4,800 patients without congenital heart disease, but 28 times more prevalent in patients with congenital heart defects^[3]^.

The association between systemic venous return variations and TOF was found in about 15% of 973 angiographies reviewed by Pandey et al.^[4]^ Persistence of left superior vena cava (11.5%) was the majority, and anomalous course of the brachiocephalic vein was seen in 4% of cases.

Kahkouee et al.^[5]^ focused on ALBCV evaluation of > 1,300 patients and found 22 cases. When present, this anatomic variation was associated with TOF in more than half of the cases. It is worth mentioning, however, that these authors have accounted the retroaortic course of the LBCV.

In this specific case, ALBCV has retrotracheoesophageal course, with appearance of the architectural garland configuration^[6]^. The incidence of this particular case is not known since most studies have considered all variations of LBCV as aberrant.

The anomalous brachiocephalic vein is rare, however, knowing the existence of these anomalies associated to TOF is relevant when undergoing invasive procedures and surgical approach. Differing from LVC persistence, in this setting, no modifications in venous canulation for cardiopulmonary bypass were necessary and they did not affect the surgical technique or final postoperative result.

**Table:** 

Abbreviations, Acronyms & Symbols	
ALBCV	= Anomalous left brachiocephalic vein
LBCV	= Left brachiocephalic vein
LVC	= Left vena cava
TOF	= Tetralogy of Fallot
